# The meniscal ossicle associated with medial meniscus posterior root tear

**DOI:** 10.1259/bjrcr.20210243

**Published:** 2022-07-01

**Authors:** Yusuf Omar Qalib, Yicun Tang, Huading Lu

**Affiliations:** 1 Department of Orthopaedics, The Fifth Affiliated Hospital of Sun Yat-Sen University, Zhuhai, Guangdong, China

## Abstract

Ossicle of the meniscus is an uncommon discovery often misdiagnosed as a loose body, which may lead to intermittent knee discomfort. We present a rare case of meniscal ossicle accompanied by the medial meniscus posterior root tear. A 46-year-old female experienced intermittent left knee pain and after coming to the hospital was diagnosed with a meniscal ossicle. The patient underwent arthroscopic ossicle resection followed by meniscal root repair. The patient had not experienced any complications post-operatively and remains asymptomatic 8 months after the surgery. The purpose of this article is to expand the knowledge of meniscal ossicle and provide a broaden review of its diagnosis and repair.

## Introduction

Meniscus ossification is an extremely rare disorder.^1^ The pathogenesis is still unclear. The researchers proposed three different theories as to the etiology of meniscal ossicle, which are as follows: degenerative, congenital and post-traumatic.^2^ A meniscal ossicle is often associated with various meniscal tears, of which tear of the posterior root of the medial meniscus (PRMM) is the most common.^3^ Pain is the most common symptom among the patients presenting with meniscal ossicle and results from mass effect or associated tear. The rate of misdiagnosis is quite high due to its presentation on radiologic imaging modalities. On X-ray scans, meniscal ossicle can be mistaken for a loose body, whereas on MRI it can be misdiagnosed as a tear or chondrocalcinosis due to increased signal sensitivity.^4^ Hereby, we present a case of meniscal ossicle associated with the posterior root of medial meniscus tear with emphasis on radiologic imaging and arthroscopic findings.

## Case report

**Figure 1.**
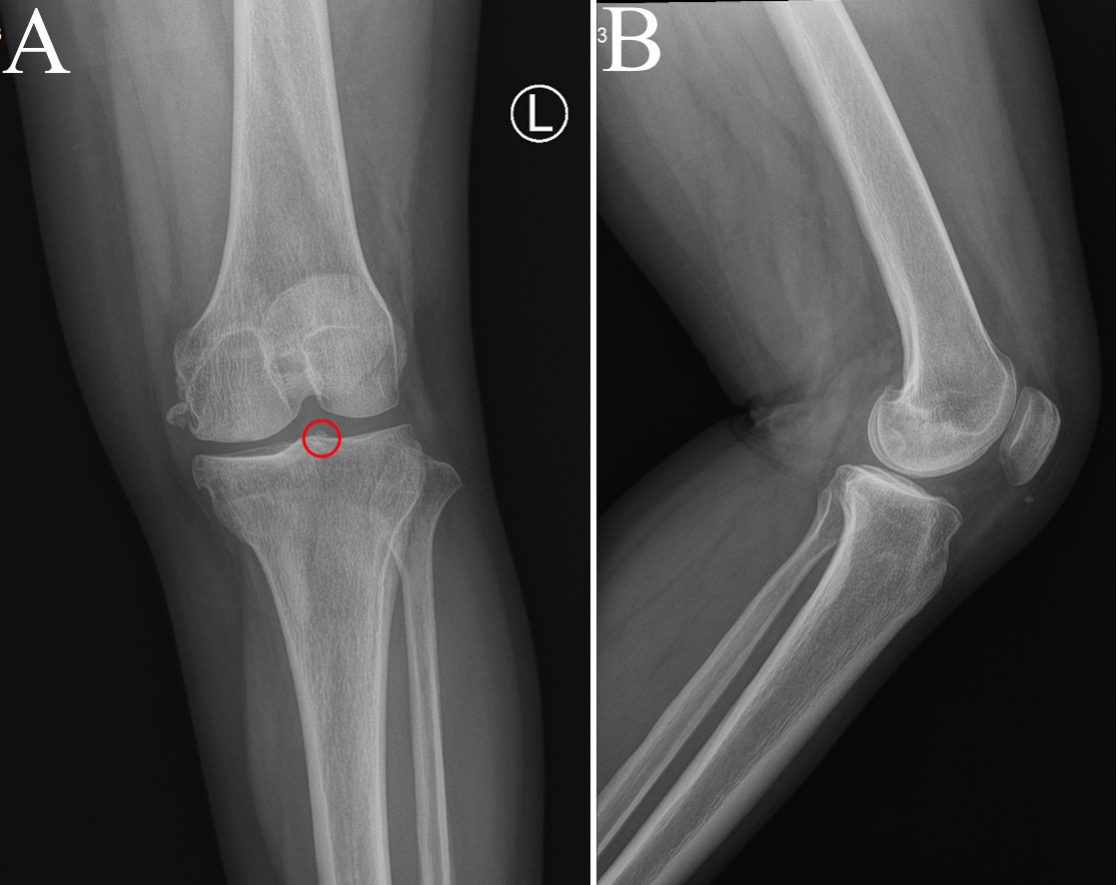
X-ray scan showing meniscal ossicle (circle).

**Figure 2.**
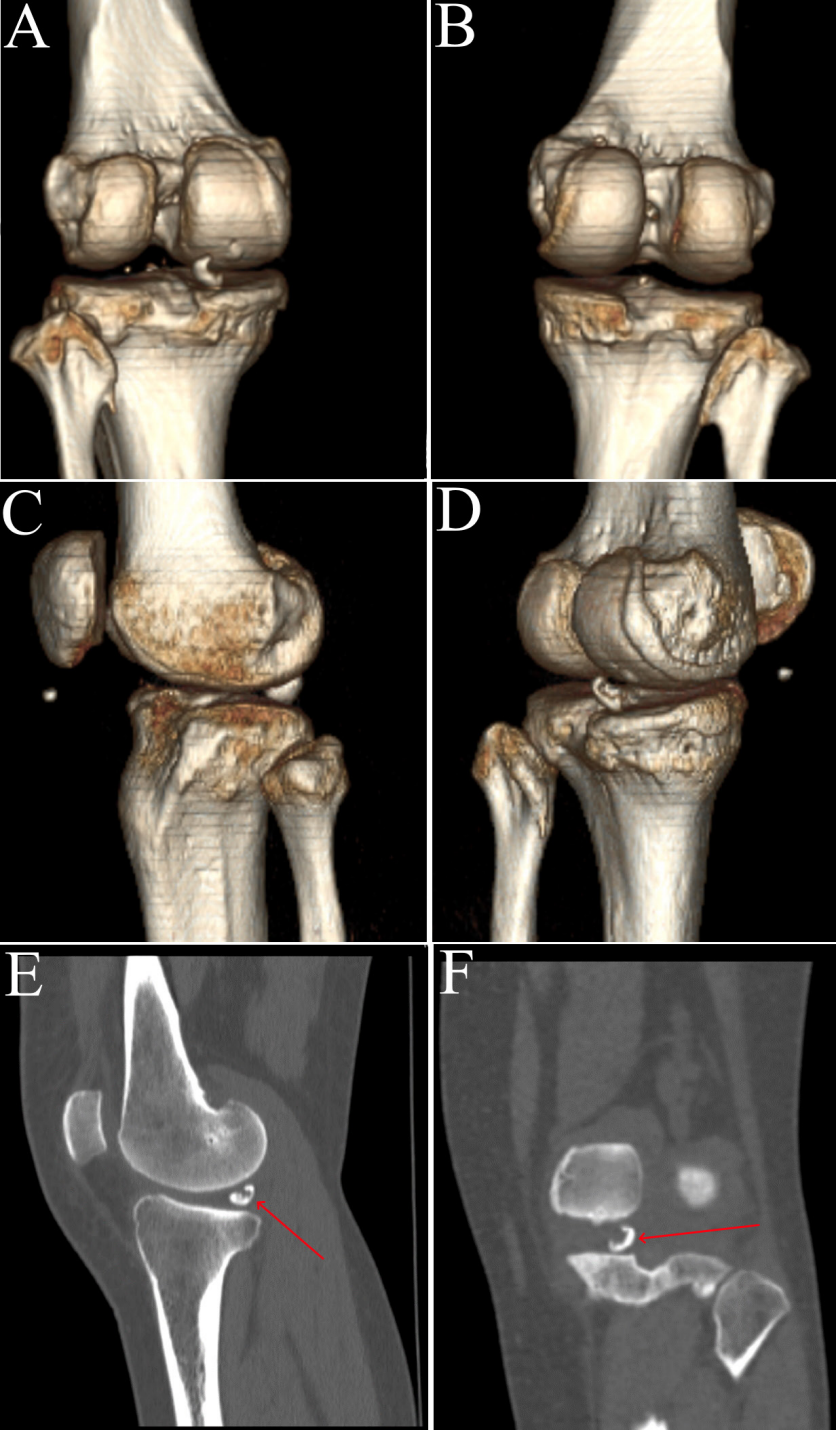
Sagittal CT scan (**A-**D) 3D model generated from CT scan showing meniscal ossicle (**E-**F) meniscal ossicle (straight arrow). 3D, three-dimensional.

**Figure 3.**
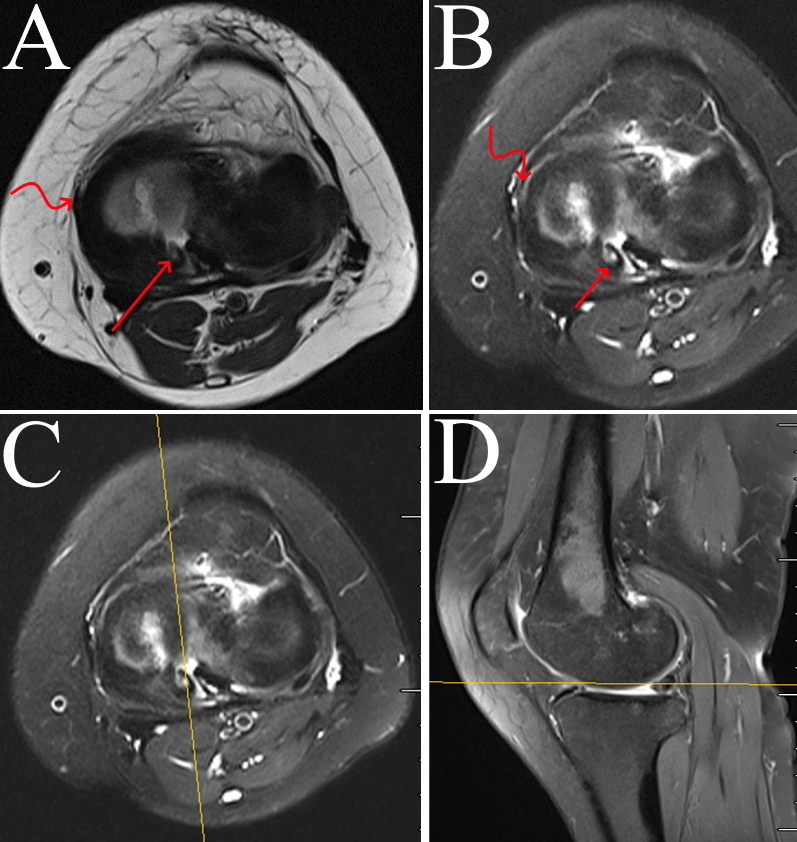
(A, B) Axial MRI scan showing medial meniscus (curved arrow) and meniscal ossicle (straight arrow) (**C, **D) location of the meniscal ossicle on axial and sagittal MRI scans.

**Figure 4.**
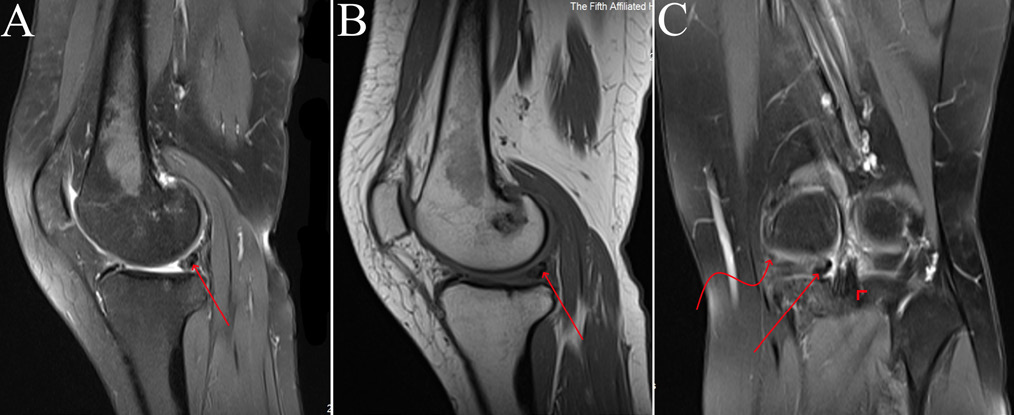
Sagittal MRI scan showing meniscal ossicle (straight arrow), medial meniscus (curved arrow) and posterior cruciate ligament (arrowhead). Note cancellous bone “inside” and cortical bone “outside” of the meniscal ossicle on (**A).**

A 46-year-old female presented to our outpatient clinic with intermittent chronic left knee pain of unknown origin for 1 year. In the last month, the pain was aggravated by walking but alleviated after rest. The patient reported no recent history of trauma, surgeries, etc. Physical examination revealed medial joint line tenderness that did not affect the movements, no signs of ligamentous laxity or interlocking knee joint were observed. Conventional anteroposterior radiography of the left knee showed a small round bony fragment projecting in the posterior aspect of the intercondylar eminence, which was initially identified as a loose body by a radiologist (Figure 1). CT scan revealed a high-density bone mass in the posteromedial side of the knee joint (Figure 2). MRI demonstrated a small, well-defined hyperintense lesion lying within the posterior horn of the medial meniscus (PHMM), isointense to the bone marrow, which is suggestive of meniscal ossicle (Figures 3 and 4). No osteochondral lesions were observed. Considering clinical and imaging findings, the diagnosis of meniscal ossicle and PRMM tear were made, so surgery to repair the PRMM and resect ossicle were suggested to the patient.

**Figure 5.**
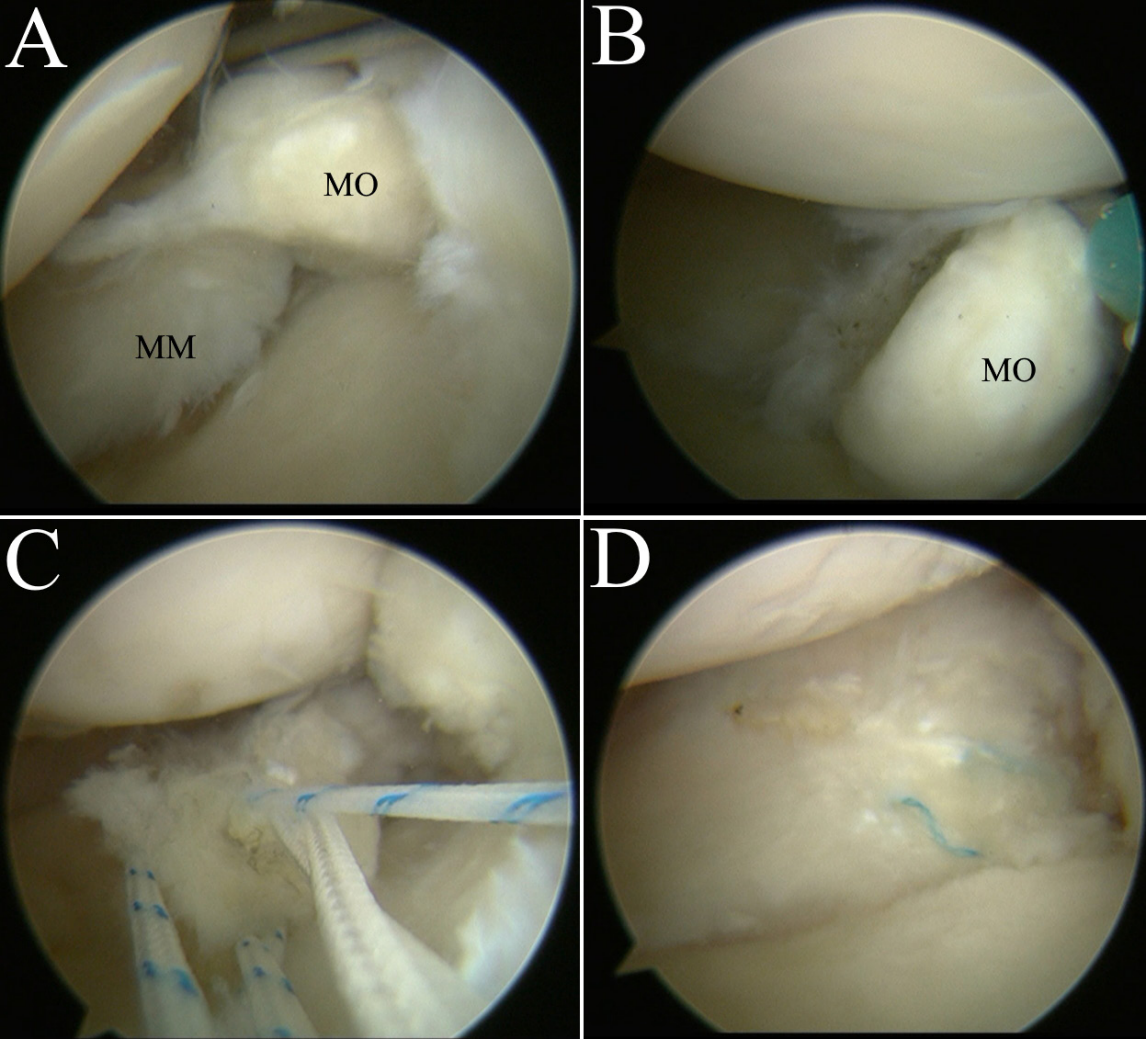
Arthoscopy images (**A, B**) MM and MO are visualized (C) after resecting meniscal ossicle, a root tear was identified and repaired with three super strong sutures using transtibial pullout technique (D) repaired medial meniscal posterior root. MO, meniscal ossicle; MM, medial meniscus.

Arthroscopy revealed a meniscal ossicle of a diameter of 1 cm adjacent to the PHMM (Figure 5A and B), PRMM tear and a small injury (1 cm^2^) of articular cartilage of the medial femoral condyle. Following identification and resection of the meniscal ossicle, PRMM tear was repaired via transtibial pullout technique (Figure 5C and D). After that, we exposed the damaged surface of the medial femoral condyle. Kirschner wires were used to drill into the damaged cartilage allowing to begin microfracture surgery. No abnormalities were observed at the end of the surgery. The patient was sent back to the ward after surgery and soon discharged from the hospital. She did not complain of any discomfort after surgery and during follow-up (the last one took place 8 months post-operatively).

## Discussion

Among existing theories of the pathogenesis of meniscal ossicles, post-traumatic theory is the most widely accepted and most commonly found in the relevant literature.^5–7^ An injury may lead to heterotopic ossification, metaplasia and osseous avulsion, contributing to meniscal ossicle development.^3^ However, our patient did not report any history of trauma.

A recent research not only supported findings stated in previous articles that PRMM is the most frequent site of occurrence of meniscal ossicles, but also that progression of meniscal ossicles may be a result of PRMM tear.^8^ In addition, PRMM may be at an increased risk of meniscal ossicles formation due to higher blood supply compared to other areas of medial meniscus.^9^

According to the latest study, the vast majority of patients complain of pain, which is reported by 89% of the patients. Increased laxity or locking is also seen, accounting for 9% of the cases.^8^ As for the diagnosis of medial ossicles on radiologic imaging modalities, X-ray is associated with a high rate of misdiagnosis, which occurred in our case as well, whereas MRI remains the gold-standard. The latter can be effectively and efficiently used in the process of diagnosis-making, but its sensitivity varies among the radiologists. Nguyen et al mentioned that meniscal ossicle can be diagnosed as a signal isointense to the bone marrow on sagittal PD-weighted MRI scan.^4^ A recent study revealed that although *T*_1_ weighted spin-echo scans show meniscal ossicles as having high intensity signal, meniscal ossicles appearing as low signal intensity are not rare. Notably, this study also concluded that prevalence of meniscal ossicle is associated with PRMM lesion on MRI.^10^

Asymptomatic patients do not require a surgical intervention. However, when symptoms or other tears are present, ossicle removal is the suggested approach. Patients that were diagnosed with meniscal ossicles and MMRT tear and received surgical treatment had a significantly higher outcome score compared to patients that received only conservative treatment.^8^ Moreover, orthopaedicians should always keep in mind the possible risk of progression of arthritis in such patients.^8,11^ A pullout repair can be used to repair MMRT tear. This technique can restore load-absorbing function of the medial meniscus and reduce meniscal extrusion thus reduce risk, thus, reduce the development of osteoarthritis.^12^

In conclusion, meniscal ossicle is rarely seen in clinics. It is of outmost importance for every orthopedician and radiologist to be familiar with this entity, its diagnosis and treatment. Since our patient was symptomatic and was diagnosed with underlying PRMM lesion, a surgery was immediately suggested to her. The patient showed good recovery post-operatively and did not complain of any symptoms.

## Learning points

1. A meniscal ossicle is rarely seen in clinics, can occur without a history of trauma, and can be misdiagnosed on X-ray as a loose body, whereas MRI remains the gold-standard.
2. A pre-operative diagnosis of meniscal ossicle can help every orthopedician and radiologist to be familiar with this entity.
